# Supplementary material for: Synthetic control of correlated disorder in UiO-66 frameworks
Source: Nat Commun. 2023 Oct 31;14:6962. doi: 10.1038/s41467-023-41936-w (PMC10618523; doi:10.1038/s41467-023-41936-w)
Supplement: Supplementary file 1 — Supplementary Information [file 41467_2023_41936_MOESM1_ESM.pdf]

Supplementary Information

Synthetic control of correlated disorder in UiO-66 frameworks

Tatay et al.

Contents

1 Reported Experimental Conditions **S-3**

2 Systematic exploration of temperature and chemical space (L/M/Mod) **S-5**

2.1 Experimental conditions used for Series A, B and C S-5

2.2 Series A S-7

2.3 TGA-MS analysis S-9

2.4 TGA analysis S-11

2.5 Series B S-12

2.6 Series C S-13

3 Effect of sample treatment and data acquisition method on PXRD profiles **S-16**

4 Effect of *reo* domain formation on framework structure and porosity **S-17**

4.1 Full cluster connectivity determination S-21

5 Imaging of defect domains with high-resolution electron microscopy **S-23**

6 Effect of defect concentration on catalytic performance **S-28**

7 Supplementary Notes **S-31**

Supplementary References **S-33**

1 Reported Experimental Conditions

Based in the excellent bibliographic survey by Feng et *al.*,[S1] in Table 1 we report a detailed summary of the conditions used by different authors to obtain UiO-66 structures with missing cluster type of defects.

Supplementary Table 1: Summary of the conditions used by different authors to obtain UiO-66 structures with missing cluster type of defects. When multiple ratios were reported by the same authors, we have only summarized the highest and lowest ratios

L[a]	L [mmol]	L [mg]	salt	M [mmol]	M [mg]	Mod1[b]	Mod1 [mmol]	Mod1[c] [mL,g]	Mod2[b]	Mod2 [mmol]	Mod2 [mL]	S1	S1 [mL]	S2	S2 [mmol]	S2 [mL]	t [h]	T [°C]	L/M	Mod/L	Ref[d]
$BDC-NH_2$	0.2	37	$ZrCl_4$	0.2	48	-	0.0	0.0	-	-	-	DMF	6.0	H_2O	-	0.01	24	120	1.0	0.0	13
$BDC-NH_2$	0.2	37	$ZrCl_4$	0.2	48	AA	200.0	11.4	-	-	-	DMF	6.0	H_2O	-	0.01	24	120	1.0	970.9	13
BDC	16.3	2700	$ZrCl_4$	16.2	3780	HCl	34.6	2.9	-	-	-	DMF	97.4	-	-	-	20	100	1.0	2.1	22
BDC	16.3	2700	$ZrCl_4$	16.2	3780	HCl	34.6	2.9	-	-	-	DMF	97.4	-	-	-	20	160	1.0	2.1	22
BDC	16.3	2700	$ZrCl_4$	16.2	3780	HCl	34.6	2.9	-	-	-	DMF	97.4	-	-	-	20	220	1.0	2.1	22
BDC	32.4	5390	$ZrCl_4$	16.2	3780	HCl	34.6	2.9	-	-	-	DMF	97.4	-	-	-	20	100	2.0	1.1	22
BDC	32.4	5390	$ZrCl_4$	16.2	3780	HCl	34.6	2.9	-	-	-	DMF	97.4	-	-	-	20	160	2.0	1.1	22
BDC	32.4	5390	$ZrCl_4$	16.2	3780	HCl	34.6	2.9	-	-	-	DMF	97.4	-	-	-	20	220	2.0	1.1	22
BDC	0.3	166	$HfCl_4$	0.3	96	FA	26.5	1.0	-	-	-	DMF	4.0	-	-	-	48	120	3.3	26.5	24
BDC	0.3	50	$HfCl_4$	0.3	96	FA	63.6	2.4	-	-	-	DMF	4.0	-	-	-	48	120	1.0	212.1	24
BDC	0.1	8	$HfCl_4$	0.3	96	FA	26.5	1.0	-	-	-	DMF	4.0	-	-	-	48	120	0.2	530.1	24
BDC	1.4	230	ZrOCl2	1.4	460	FA	70.0	2.7	-	-	-	DMF	23.0	-	-	-	24	120	1.0	50.0	25
BDC	0.3	42	$HfCl_4$	0.3	96	FA	53.0	2.0	-	-	-	DMF	4.0	-	-	-	24	150	0.8	212.1	26
BDC	0.5	83	$HfCl_4$	0.3	96	FA	53.0	2.0	-	-	-	DMF	4.0	-	-	-	24	150	1.7	106.0	26
BDC	2.3	379	$ZrCl_4$	1.6	380	-	0.0	0.0	-	-	-	DMF	30.0	H_2O	11.1	0.2	12	100	1.4	4.9	41
BDC	2.3	379	$ZrCl_4$	1.6	380	-	0.0	0.0	-	-	-	DMF	30.0	H_2O	111.1	2.0	12	100	1.4	48.7	41
BDC	0.5	83	$ZrCl_4$	0.5	117	FA	12.5	0.5	-	-	-	DMF	30.0	-	-	-	24	120	1.0	25.0	40
BDC	0.5	83	$ZrCl_4$	0.5	117	FA	75.0	2.8	-	-	-	DMF	30.0	-	-	-	24	120	1.0	150.0	40
BDC	0.5	83	$ZrCl_4$	0.5	117	AA	12.5	0.7	-	-	-	DMF	30.0	-	-	-	24	120	1.0	25.0	40
BDC	0.5	83	$ZrCl_4$	0.5	117	AA	75.0	4.3	-	-	-	DMF	30.0	-	-	-	24	120	1.0	150.0	40
BDC	0.5	83	$ZrCl_4$	0.5	117	FA	50.0	1.9	-	-	-	DMF	30.0	-	-	-	24	100	1.0	100.0	40
BDC	1.0	167	ZrOCl2	1.0	322	TFA	10.0	0.8	HCl	1	0.1	DMF	10.2	-	-	-	72	120	1.0	10.0	47
BDC	1.0	174	$ZrCl_4$	1.0	233	-	0.0	0.0	-	-	-	DMF	20.0	-	-	-	24	120	1.0	0.0	47
BDC	1.4	233	ZrOCl2	1.4	460	FA	70.0	2.7	-	-	-	DMF	23.0	-	-	-	24	120	1.0	50.0	47
$BDC-NH_2$	1.5	268	$ZrCl_4$	1.1	250	HCl	121.0	10.0	-	-	-	DMF	50.0	-	-	-	24	200	1.4	81.6	56
$BDC-NH_2$	1.5	268	$ZrCl_4$	1.1	250	HCl	121.0	10.0	-	-	-	DMF	50.0	-	-	-	24	180	1.4	81.6	56
$BDC-NH_2$	1.5	268	$ZrCl_4$	1.1	250	HCl	121.0	10.0	-	-	-	DMF	50.0	-	-	-	24	160	1.4	81.6	56
$BDC-NH_2$	1.5	268	$ZrCl_4$	1.1	250	HCl	121.0	10.0	-	-	-	DMF	50.0	-	-	-	24	120	1.4	81.6	56
$BDC-NH_2$	1.5	268	$ZrCl_4$	1.1	250	HCl	121.0	10.0	-	-	-	DMF	50.0	-	-	-	24	80	1.4	81.6	56
$BDC-NH_2$	1.5	268	$ZrCl_4$	1.1	250	-	0.0	0.0	-	-	-	DMF	50.0	H_2O	-	0.15	24	200	1.4	0.0	56
BDC	0.8	129	$ZrCl_4$	0.8	180	-	0.0	0.0	-	-	-	DMF	30.0	H_2O	0.77	0.014	48	120	1.0	0.0	57
BDC	2.0	332	$ZrCl_4$	2.0	466	BA	20.0	2.4	-	-	-	DMF	36.0	H_2O	2.0	0.036	48	120	1.0	10.0	57
BDC	1.9	307	$ZrCl_4$	1.9	431	FA	67.0	2.5	-	-	-	DMF	50.0	H_2O	5.6	0.1	72	120	1.0	36.2	57
BDC	3.2	532	$ZrCl_4$	3.2	746	HCl	302.5	25.0	-	-	-	DMF	75.0	-	-	-	24	120	1.0	94.5	58

[a] L: BDC = benzene-1,4-dicarboxylic acid, $BDC-NH_2$ = 2-aminobenzene-1,4-dicarboxylic acid, [b] Modulator: AA = acetic acid, TFA = trifluoroacetic acid, FA = formic acid, BA = benzoic acid, [c] HCl, AA, TFA and FA amounts expressed as milliliters, BA amount expressed as grams, [d] References according to those used in maintext Figure 1.

2 Systematic exploration of temperature and chemical space (L/M/Mod)

2.1 Experimental conditions used for Series A, B and C

M = HCl_4, L = BDC, Mod = Formic Acid

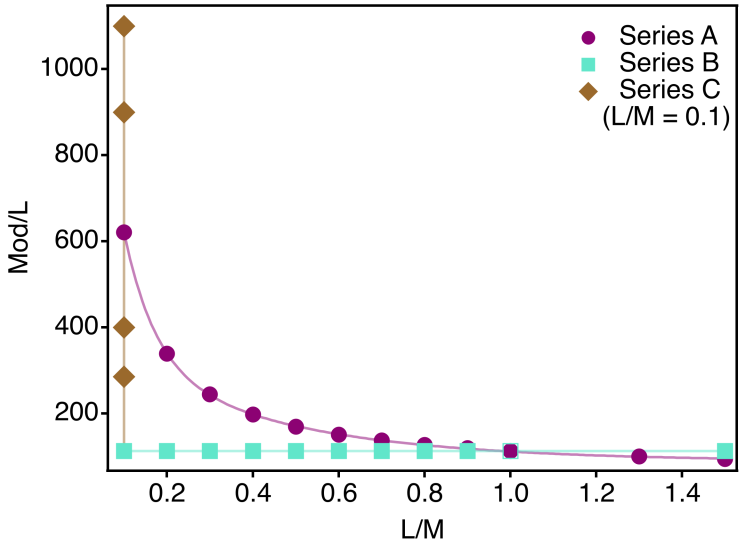

Supplementary Figure 1: L/M and Mod/L used for the preparation of Series A, B and C (L/M=0.1) samples. M+L = 0.6 and V = 5 mL.

Supplementary Table 2: Model conditions used for Series A samples.

ratio L/M	Mod/L	M+L	M [mg]	M [mmol]	L [mg]	L [mmol]	Mod [mL]	Mod [mmol]	DMF [mL]	V [mL]
1.50	94.06	0.60	**78.44**	0.24	**61.09**	0.36	1.00	33.86	4.00	5.00
1.3	99.8	0.6	**85.3**	0.3	**57.5**	0.3	1.0	33.9	4.0	5.0
1.0	112.9	0.6	**98.1**	0.3	**50.9**	0.3	1.0	33.9	4.0	5.0
0.9	119.1	0.6	**103.2**	0.3	**48.2**	0.3	1.0	33.9	4.0	5.0
0.8	127.0	0.6	**108.9**	0.3	**45.3**	0.3	1.0	33.9	4.0	5.0
0.7	137.1	0.6	**115.4**	0.4	**41.9**	0.2	1.0	33.9	4.0	5.0
0.6	150.5	0.6	**122.6**	0.4	**38.2**	0.2	1.0	33.9	4.0	5.0
0.5	169.3	0.6	**130.7**	0.4	**33.9**	0.2	1.0	33.9	4.0	5.0
0.4	197.5	0.6	**140.1**	0.4	**29.1**	0.2	1.0	33.9	4.0	5.0
0.3	244.5	0.6	**150.8**	0.5	**23.5**	0.1	1.0	33.9	4.0	5.0
0.2	338.6	0.6	**163.4**	0.5	**17.0**	0.1	1.0	33.9	4.0	5.0
0.1	620.8	0.6	**178.3**	0.5	**9.3**	0.1	1.0	33.9	4.0	5.0

Supplementary Table 3: Model conditions used for Series B samples.

ratio L/M	Mod/L	M+L	M [mg]	M [mmol]	L [mg]	L [mmol]	Mod [mL]	Mod [mmol]	DMF [mL]	V [mL]
1.5	112.9	0.6	**78.4**	0.2	**61.1**	0.4	1.2	40.6	3.8	5.0
0.9	112.9	0.6	**103.2**	0.3	**48.2**	0.3	0.9	32.1	4.1	5.0
0.8	112.9	0.6	**108.9**	0.3	**45.3**	0.3	0.9	30.1	4.1	5.0
0.7	112.9	0.6	**115.4**	0.4	**41.9**	0.2	0.8	27.9	4.2	5.0
0.6	112.9	0.6	**122.6**	0.4	**38.2**	0.2	0.7	25.4	4.3	5.0
0.5	112.9	0.6	**130.7**	0.4	**33.9**	0.2	0.7	22.6	4.3	5.0
0.4	112.9	0.6	**140.1**	0.4	**29.1**	0.2	0.6	19.3	4.4	5.0
0.3	112.9	0.6	**150.8**	0.5	**23.5**	0.1	0.5	15.6	4.5	5.0
0.2	112.9	0.6	**163.4**	0.5	**17.0**	0.1	0.3	11.3	4.7	5.0
0.1	112.9	0.6	**178.3**	0.5	**9.3**	0.1	0.2	6.2	4.8	5.0

Supplementary Table 4: Model conditions used for Series C (L/M = 0.1) samples.

ratio L/M	Mod/L	M+L	**M [mg]**	M [mmol]	**L [mg]**	L [mmol]	Mod [mL]	Mod [mmol]	DMF [mL]	V [mL]
0.1	285.7	0.6	**178.3**	0.5	**9.3**	0.1	0.5	15.6	4.5	5.0
0.1	399.9	0.6	**178.3**	0.5	**9.3**	0.1	0.6	21.8	4.4	5.0
0.1	899.9	0.6	**178.3**	0.5	**9.3**	0.1	1.4	49.1	3.6	5.0
0.1	1100.0	0.6	**178.3**	0.5	**9.3**	0.1	1.8	60.0	3.2	5.0

2.2 Series A

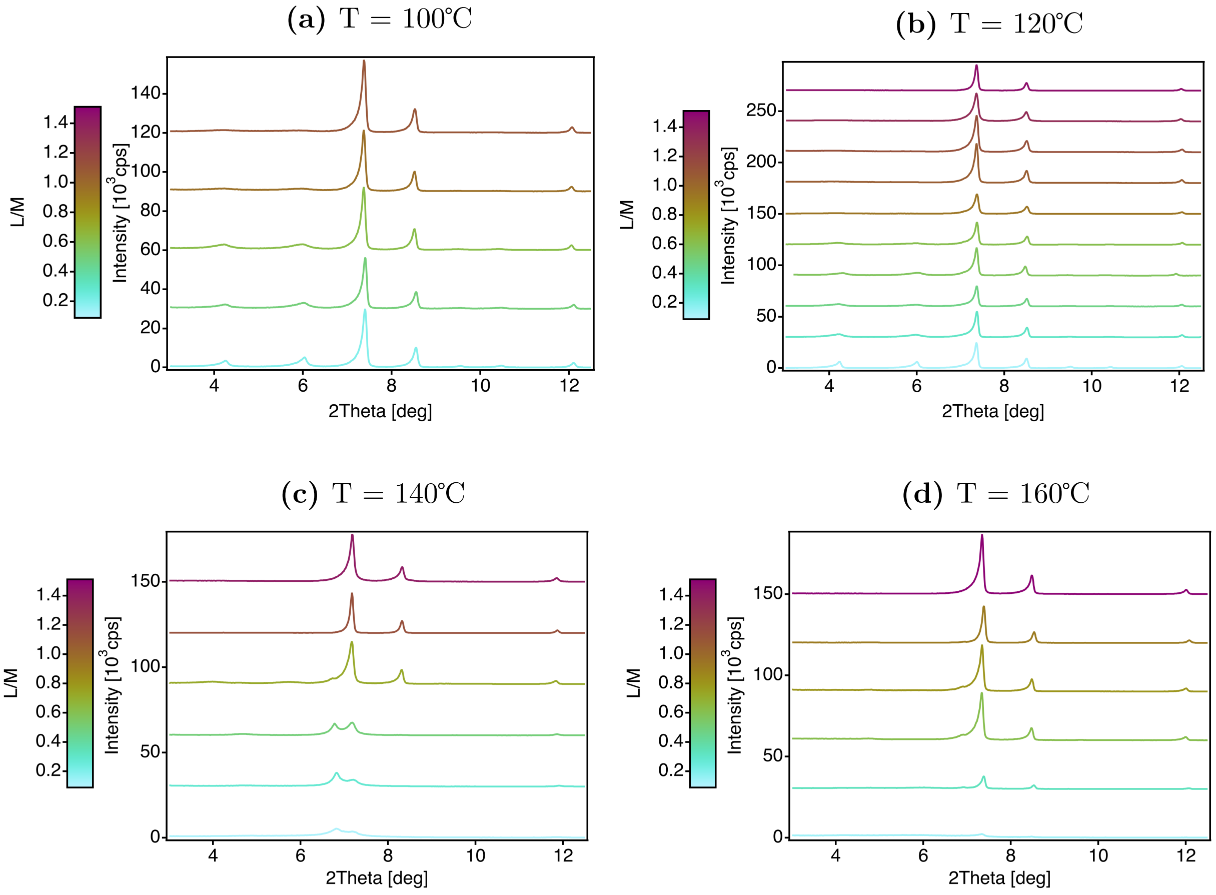

Supplementary Figure 2: PXRD in the low 2θ range as a function of the synthesis temperature and the L/M ratio for a set of selected samples.

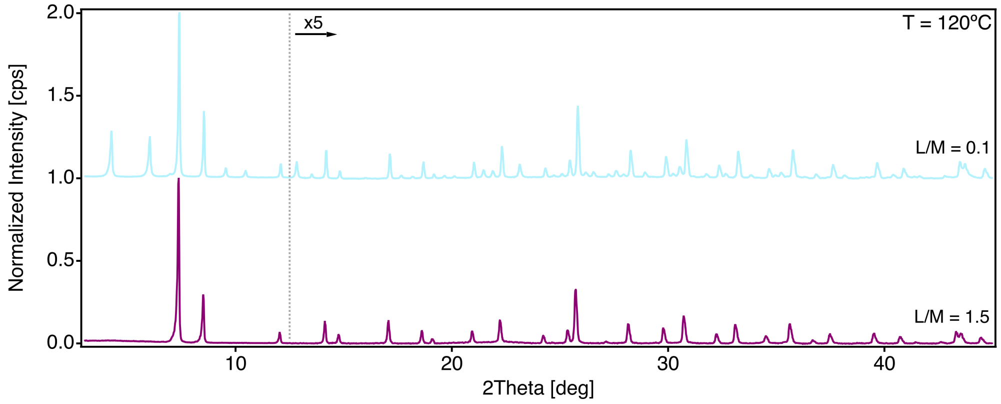

Supplementary Figure 3: PXRD(capillar) in the low 2θ range for two T = 120°C samples with L/M =1.5 and 0.1.

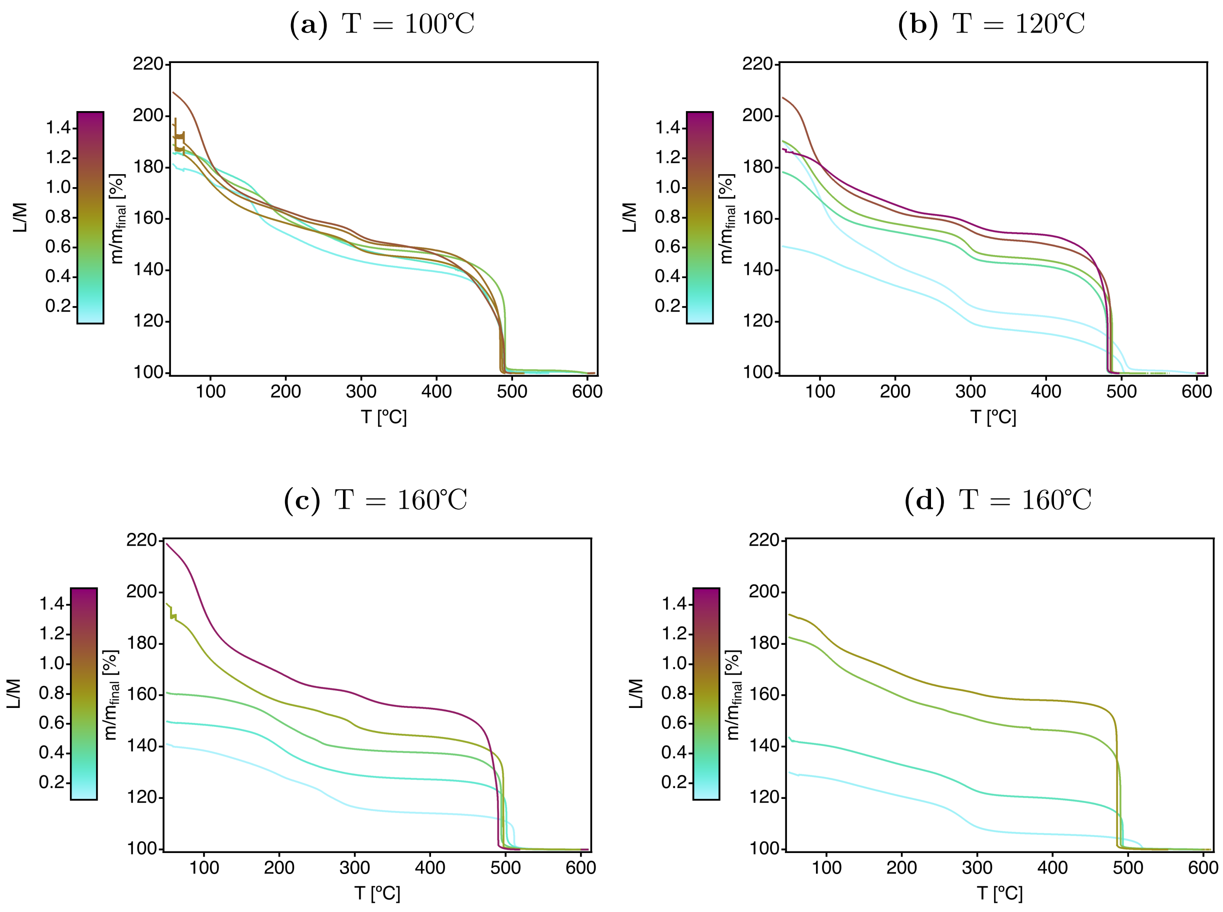

Supplementary Figure 4: TGA as function of the synthesis temperature and the L/M ratio for a set of selected samples.

2.3 TGA-MS analysis

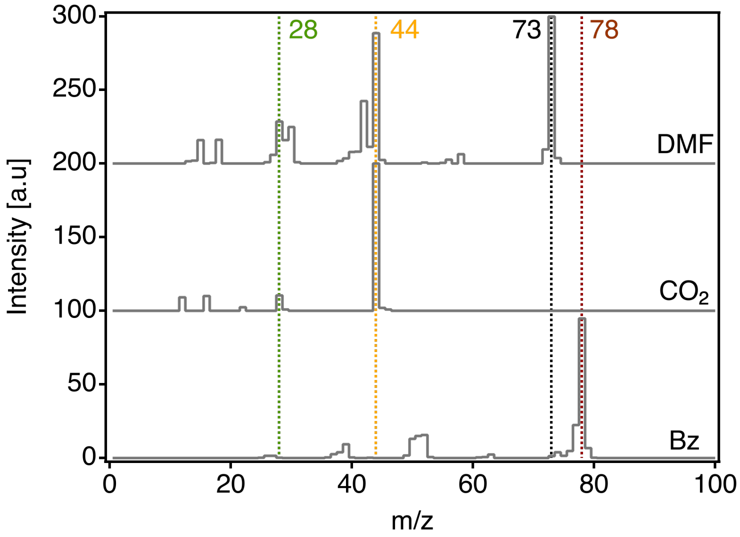

Supplementary Figure 5: MS(Electronic Ionization) spectra of reference selected compounds.[S2] m/z values of the fragments relevant to this work have been highlighted by vertical dotted lines. Bz = benzene.

According to TGA-MS data in Figure 6, the decomposition of UiO-66 samples takes place in three main temperature ranges. The first one (50 - 200℃) is characterized by the the appearance of m/z = 28, 44 and 73 signals in the MS (Figure 6d), green, yellow and black lines, respectively. This is consistent with the loss of DMF molecules and their MS fragmentation into CO, CO_2 (Figure 5). The second stage (200 - 300℃) is characterized by an increase in the m/z = 44 signal, although m/z = 28 and 73 signals can be still observed. We attribute this additional contribution to the m/z = 44 signal to the decomposition of FA molecules into CO_2. In the last stage (500 - 700℃), we see m/z = 28 and 44 signals again, and the m/z = 78 signal (wine line) appears for the first time. This is consistent with the decomposition of BDC linker into benzene (Bz) and CO_2, that in turns yields CO_2 and CO MS fragments. If we focus on the 200 - 300℃ and 500 - 700℃ regions, we can observe that the relative contribution of FA and BDC to the overall mass loss and MS profiles (Figure 6a and Figure 6d) is inversely proportional. This strongly suggest that BDC defect concentration is accompanied by an increase of FA in the structure.

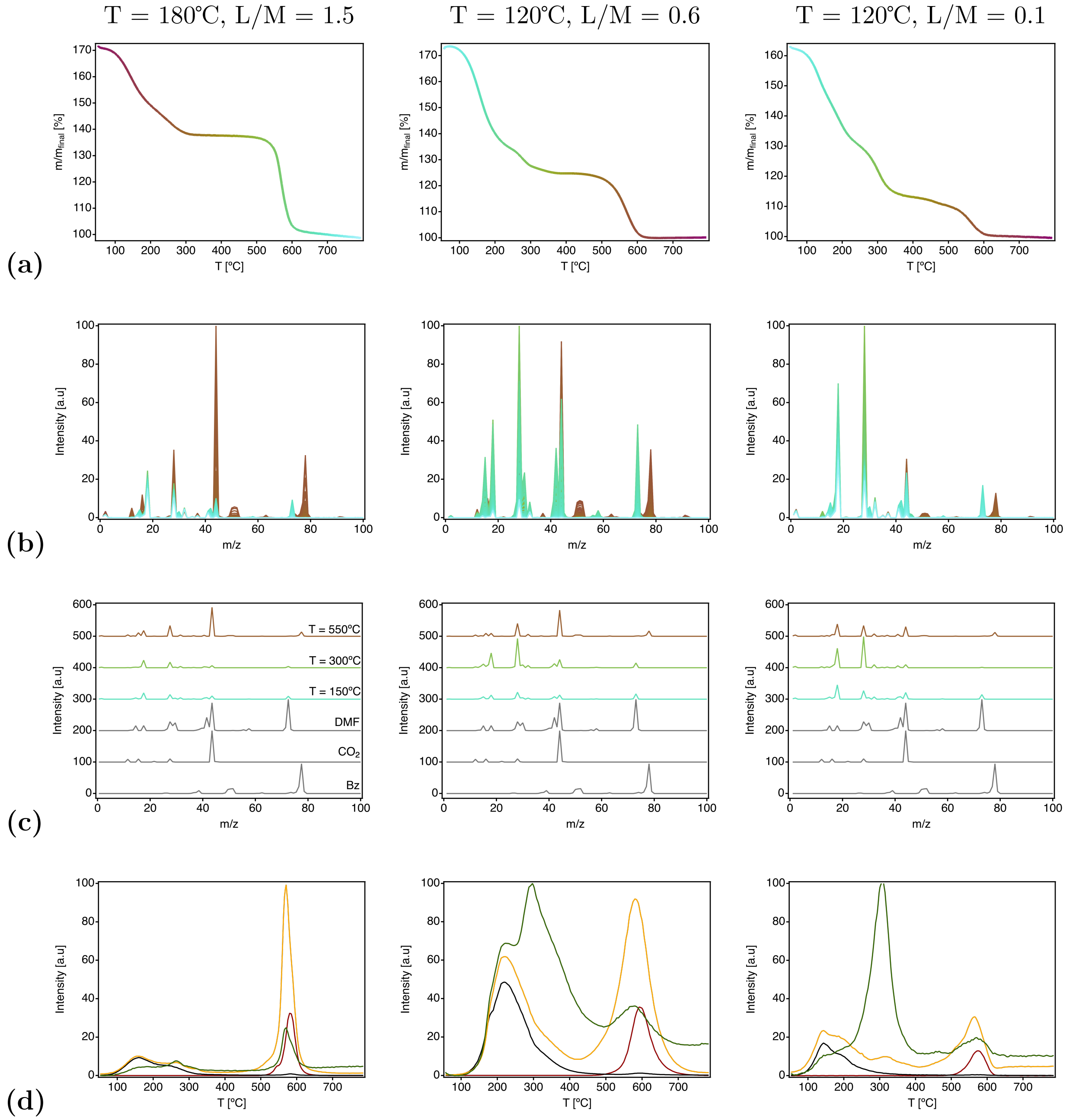

Supplementary Figure 6: TGA-MS as function of the synthesis temperature and the L/M ratio for a set of selected samples. a) TGA profile, b) Superimposed MS as a function of the temperature (T = 50 - 750°C), color code correspond to the temperatures defined by the TGA profiles in (a), c) MS spectra at T = 550, 300 and 150°C, gray lines are reference spectra for DMF, CO_2 and Bz and have been added for comparison, d) Selected m/z signals as function of the temperature.

2.4 TGA analysis

Mass changes up to 400℃ correspond to the loss of solvent and modulator molecules. This is reflected in the TGA profile as 1 - 3 mass loss steps. In this range, dehydroxylation of the $Hf_6O_4(OH)_4$ cluster to Hf_6O_6 also takes place, but is masked by solvent and modulator bigger mass losses. The final decomposition of the structure to give HfO_2 above 400℃lead to one final mass loss step.[S3] Assuming that charge for any missing BDC linker is compensated by the inclusion of two FA molecules, and that after FA decomposition it is compensated by oxygen atoms, we propose the following formulas as function the temperature:

Supplementary Table 5: Proposed formulas for UiO-66 defective and non-defective samples as a function of the temperature.

T [℃]	Non-defective	Defective
25	$[Hf_6O_4(OH)_4BDC_6]\cdot DMF_y$	$[Hf_6O_4(OH)_4BDC_{6\text{-}x}FA_{2x}]\cdot DMF_y$
400	$[Hf_6O_6BDC_6]$	$[Hf_6O_{6+x}BDC_{6\text{-}x}]$
600	HfO_2	HfO_2

For simplicity, in the following we have abbreviated m/m_{final} (mass at a given temperature/mass at 750℃) as m and we have used subindexes to refer to the temperature. According to Table 5 the number of missing linker BDC molecules (x) in the structure can be calculated as follows:

$$m_{600} = MW_{HfO_2} \cdot n_{HfO_2}$$

$$n_{MOF} = \frac{n_{HfO_2}}{6} = \frac{m_{600}}{6 \cdot MW_{HfO_2}} = \frac{100}{6 \cdot MW_{HfO_2}}$$

$$m_{450} = MW_{MOF_{450}} \cdot n_{MOF}$$

$$\mathbf{MW_{MOF_{450}}} = \frac{6 \cdot m_{450} \cdot MW_{HfO_2}}{100} = \mathbf{12.63 \cdot m_{450}}$$

$$MW_{MOF_{450}} = 6 \cdot MW_{Hf} + (6+x)MW_O + (6-x)MW_{BDC^{2-}}$$

$$\mathbf{x} = \frac{MW_{MOF_{450}} - 6(MW_{Hf} + MW_O + MW_{BDC^{2-}})}{MW_O - MW_{BDC^{2-}}} = \frac{\mathbf{2151.71 - 12.63 \cdot m_{450}}}{\mathbf{148.13}}$$

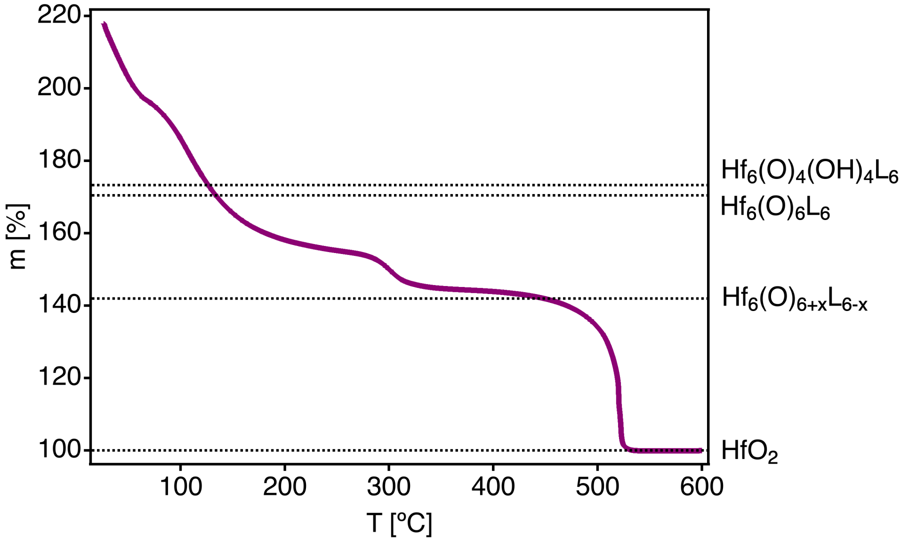

Supplementary Figure 7: TGA profile of a T = 120℃ M/L = 0.4 sample. Horizontal dotted lines are the expected masses for different expected formulas and have been added for reference.[S2] Bz = benzene.

Accordingly, sample displayed in Figure 7 with m_{450} = 142% have only 3.6 BDC linkers (x = 2.4) per cluster.

2.5 Series B

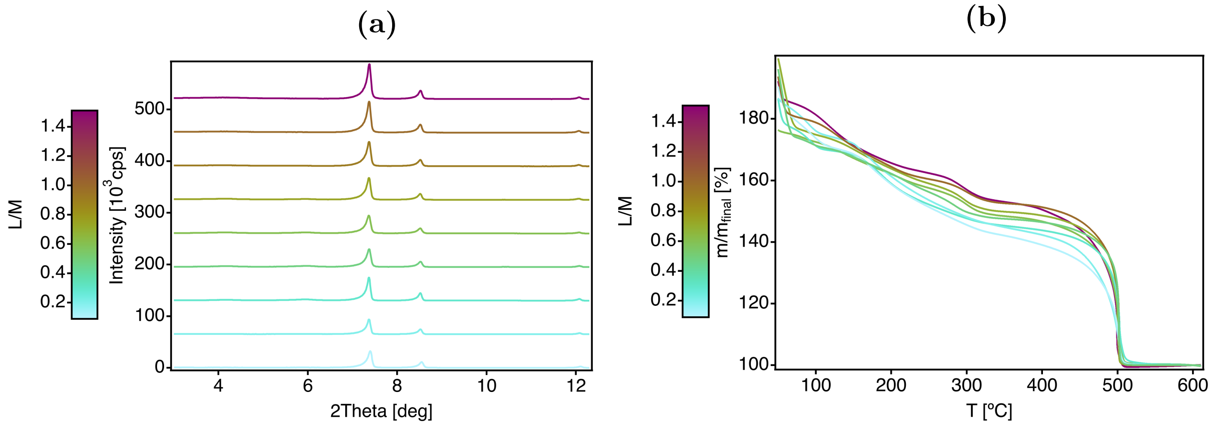

Supplementary Figure 8: a) PXRD in the low 2θ range and b) TGA as function of the L/M ratio. Synthesis temperature was T = 120℃ and Mod/L = 113 for all samples.

2.6 Series C

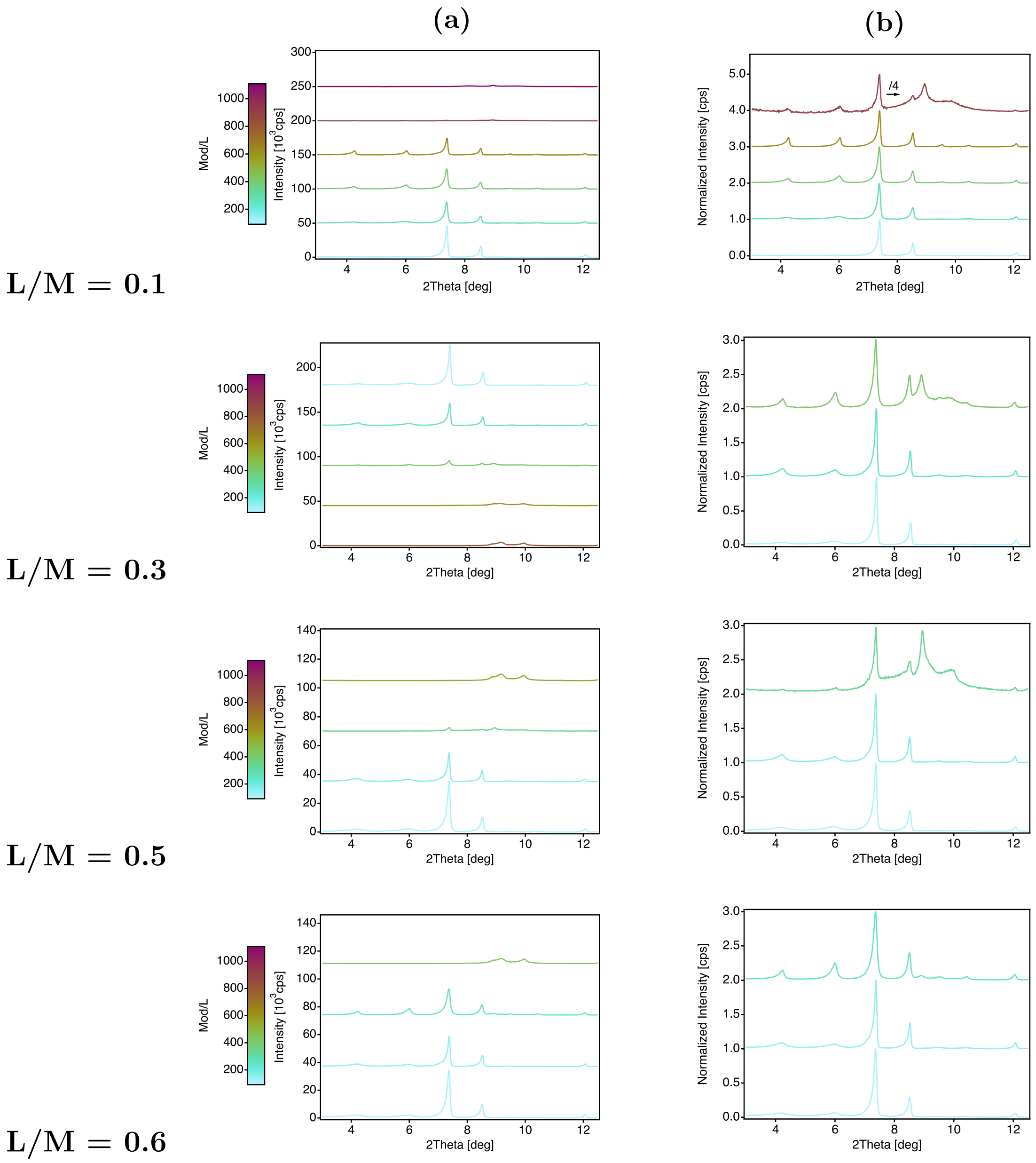

Supplementary Figure 9: a) PXRD and b) Normalized PXRD in the low 2θ range as function of the Mod/L ratio for a set of selected samples with different L/M values.

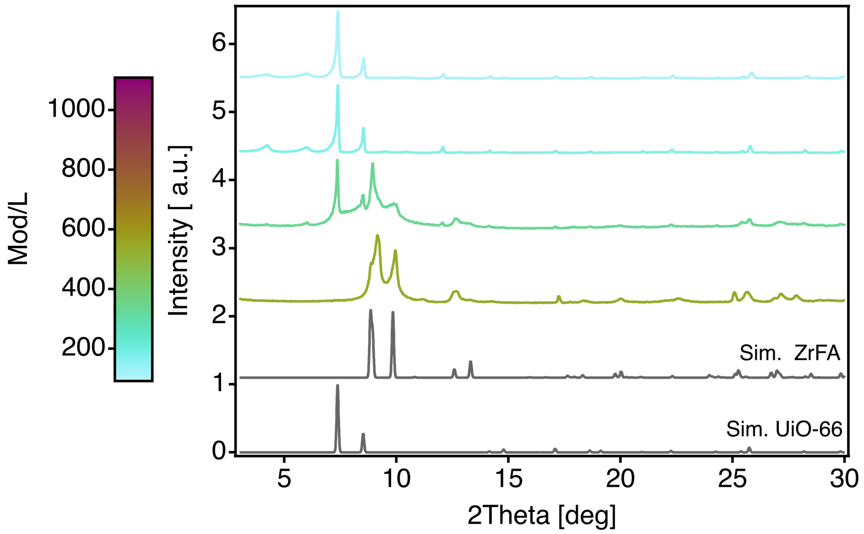

Supplementary Figure 10: Normalized PXRD as a function of the Mod/L ratio for a selected L/M = 0.5 samples synthesized at T = 120℃. Gray lines are simulated PXRD patterns for UiO-66 and ZrFA.

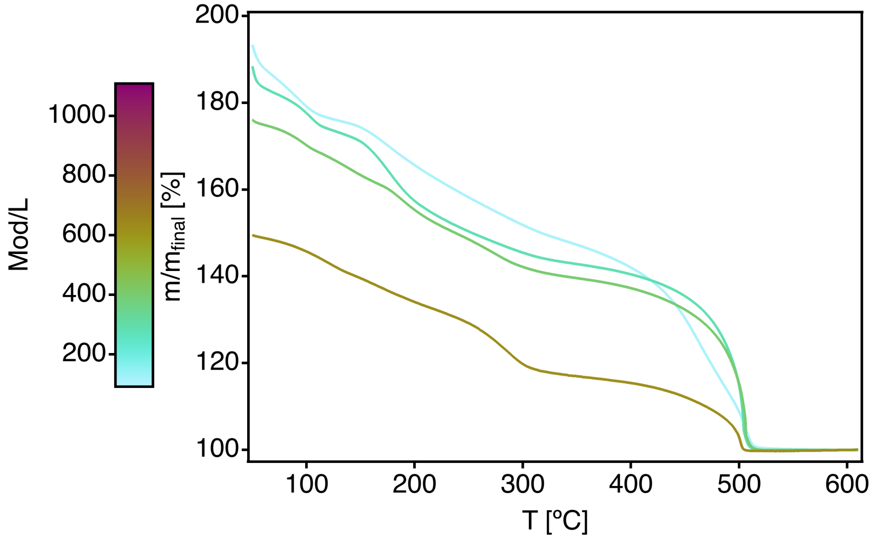

Supplementary Figure 11: TGA as a function of the Mod/L ratio. Synthesis temperature was T = 120℃ and L/M = 0.1 for all samples.

3 Effect of sample treatment and data acquisition method on PXRD profiles

It is difficult to set a lower limit for ***reo*** phase signal detection, as the ease with which supercell peaks are observed depends on the method of acquisition, the way the data are represented (e.g. linear vs. logarithmic scale) and the history of the sample. To try to illustrate this a little better we have measured exactly the same sample (L/M = 1.5, T = 120°C) using different acquisition conditions and two sample treatments.

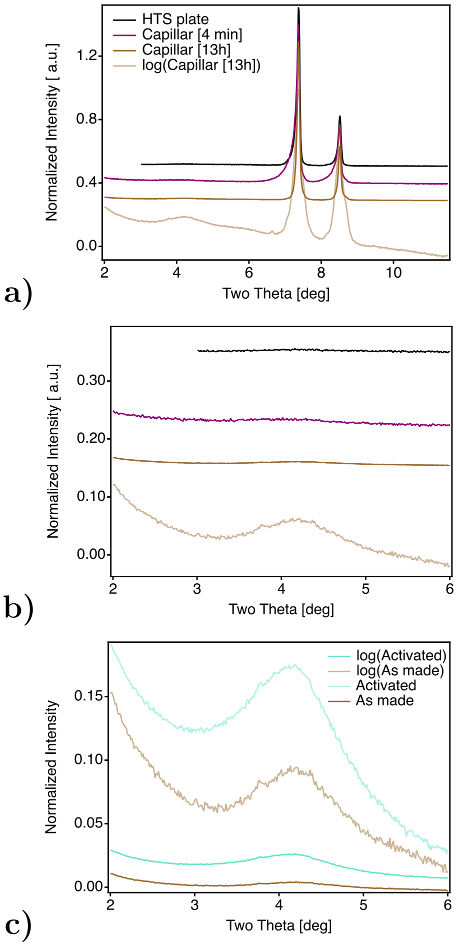

Supplementary Figure 12: Normalized PXRD profiles of the same L/M = 1.5 T = 120°C sample measured on a) HTS plate and a capillar using 4 min and 13 hours (linear and log representation) acquisition time. b) Profiles in (a) plotted using a narrower range. c) Capillar 13 h acquisition profiles measured before and after solvent exchange and activation. samples. Lines have been shifted for clarity.

4 Effect of *reo* domain formation on framework structure and porosity

Supplementary Table 6: Crystallographic Information of ***fcu*** and ***reo*** Hf-UiO-66 phases of L/M = 1.5, T = 180℃ and L/M = 0.1, T = 120℃ samples, respectively. ***reo*** Hf-UiO-66 (CCDC number 2223895, Supplementary Notes 3 to 6)

**Name**	**Non-defective UiO-66**	***reo*-UiO-66**
Empirical formula	$Hf_6O_{37.59}C_{41.57}H_{20.78}$	$Hf_6O_{38.22}C_{36}H_{20}$
Formula weight [g·mol^{-1}]	2192.59	2135.04
Temperature [K]	298	298
Crystal system	Cubic	Cubic
Space group	$Fm\bar{3}m$	$Pm\bar{3}m$
a [Å]	20.7101(1)	20.6928(3)
Volume [Å^3]	8882.72(8)	8860.5(4)
Z	4	3
Wavelength [Å]	1.540596	1.540596
2Theta range [deg]	2.0 - 105	2.0 - 105
Weight fraction [wt. %]	100	87.9(1)
Number of reflections	306	1083
Number of structural / total variables	21 / 46	41 / 93
R_p [%]	3.34	4.83
R_{wp} [%]	5.02	6.38
R_{exp} [%]	1.08	1.55
R_{Bragg} [%]	1.38	1.13
GoF	4.652	4.123

Notice that during Rietveld refinement the data is modelled as two independent crystalline phases but with overlapping peaks. As a result, when the ***reo*** fraction raises, an increasing contribution of broader ***reo*** Bragg peaks overlaps with the sharper peaks of the fcu phase, thus lowering the quality of the refinement. For high ***reo*** fractions, the peaks corresponding to the ***reo*** phase become sharper, thus reducing the width mismatch between peaks for a reduced GoF.

Supplementary Table 7: Refined lattice parameters, weight fractions (wt. %) and final goodnes-of-fit (GoF) factors obtained from the Rietveld refinement of the different UiO-66 samples.

T [℃]	L/M	a(fcu) [Å]	V(fcu) [Å^3]	a(reo) [Å]	V(reo) [Å^3]	wt.(fcu) [%]	wt.(reo) [%]	R_{Bragg}(fcu) [%]	R_{Bragg}(reo) [%]	R_{wp} [%]	GoF
180	1.5	20.7101(1)	8882.73(8)	-	-	100	-	1.380	-	5.025	4.652
120	1.5	20.7120(2)	8885.2(2)	20.661(9)	8820(11)	81.6(5)	18.3(5)	1.562	3.036	8.550	7.643
120	1	20.7032(1)	8873.9(2)	20.672(6)	8834(7)	79.8(3)	20.2(3)	2.078	2.063	5.752	5.424
120	0.8	20.6955(3)	8864.0(4)	20.665(3)	8825(4)	70.1(3)	30.9(3)	2.088	1.845	6.990	2.654
120	0.4	20.6942(3)	8862.4(5)	20.697(1)	8866(1)	42.7(5)	57.3(5)	2.737	6.540	13.186	12.765
120	0.3	20.6985(3)	8867.8(4)	20.699(1)	8868(2)	50.2(4)	49.8(4)	2.825	8.332	14.386	12.819
120	0.1	20.695(1)	8864(1)	20.6928(3)	8860.5(4)	11.9(2)	88.1(2)	1.321	1.130	6.395	4.123

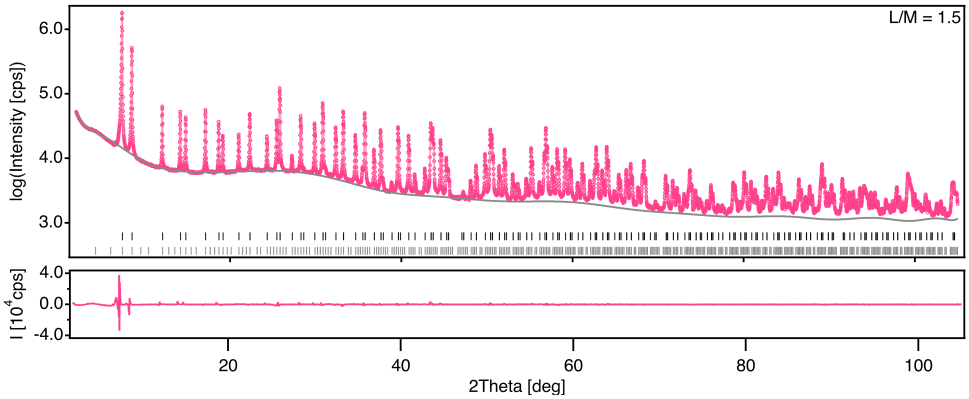

Supplementary Figure 13: Rietveld refinement of a non-defective T = 180℃, L/M = 1.5 sample. Experimental (hollow dots), calculated (solid line), difference plot [I = (I_{obs}-I_{calc})] (gray line, bottom panel) and Bragg positions (***fcu*** black ticks, ***reo*** gray ticks, bottom panel)

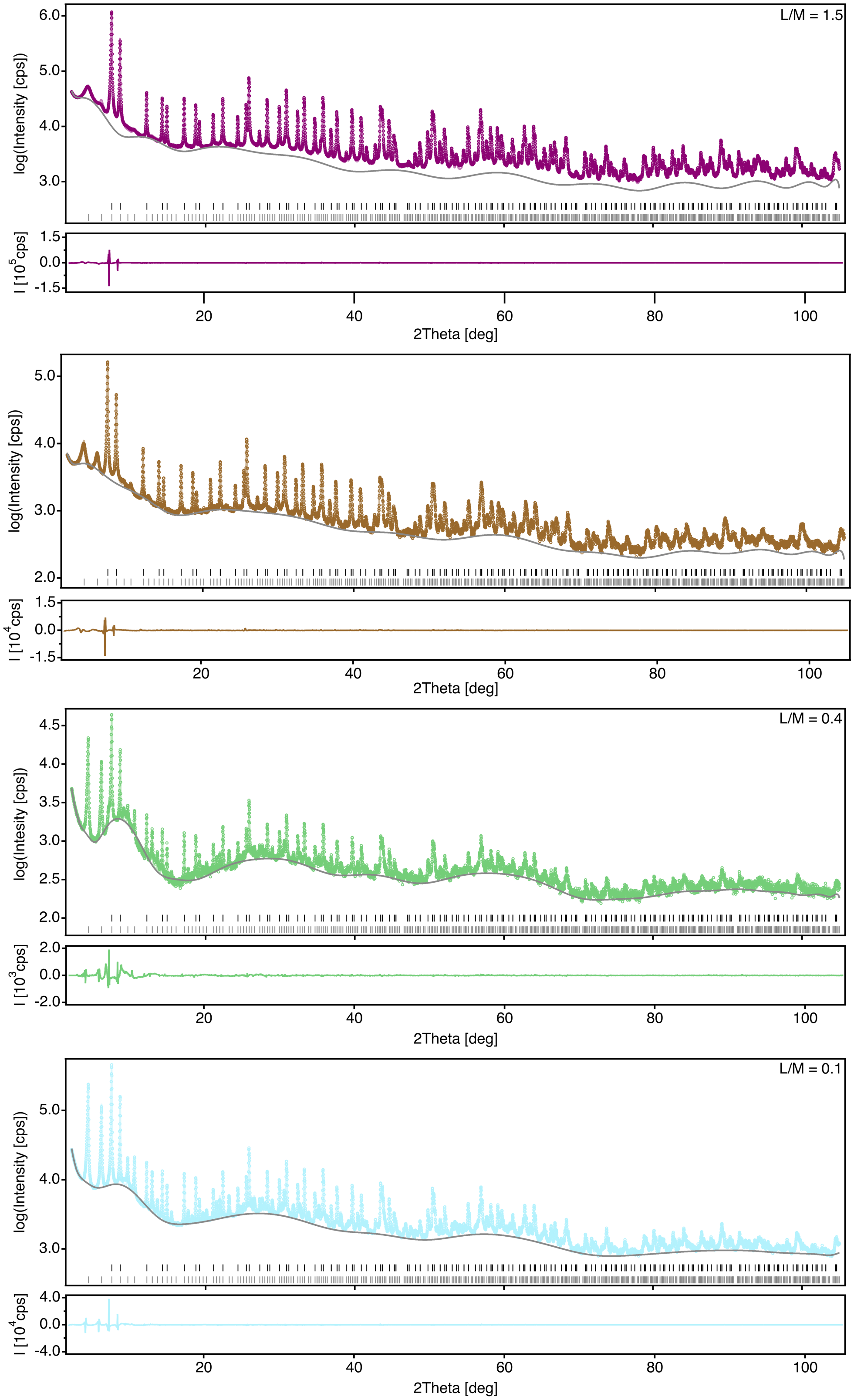

Supplementary Figure 14: Rietveld refinement as function of L/M for a selection of T = 120℃ samples. Experimental (hollow dots), calculated (solid line), difference plot [I = (I_{obs}-I_{calc})] (gray line, bottom panel) and Bragg positions (***fcu*** black ticks, ***reo*** gray ticks, bottom panel)

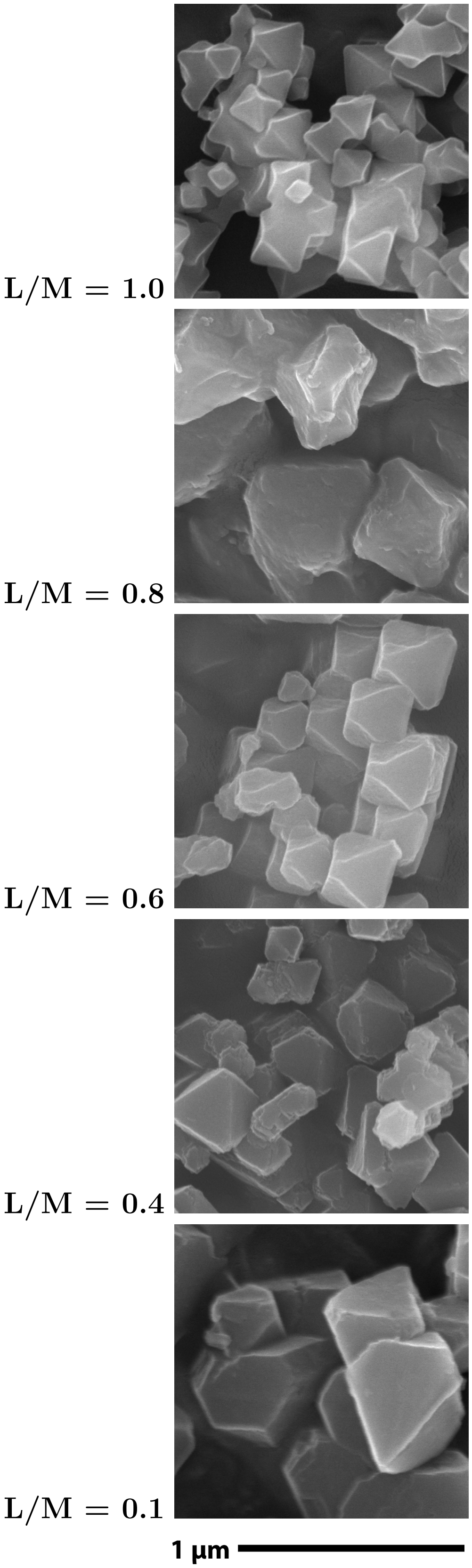

Supplementary Figure 15: SEM images as function of L/M for a selection of T = 120℃ samples.

4.1 Full cluster connectivity determination

H_2BDC solutions in $D_2O/NaHCO_3$ were used to calibrate the NMR instrument from their respective integrated signals (A):

$$C_{BDC} = \frac{52.97\ mg}{mL} \cdot \frac{A_{BDC}}{A_{D_2O}}$$

BDC %w/w content was determined using the above ^{1}H-NMR calibration and compared to TGA data. As can be seen in Figure 16, ^{1}H-NMR and TGA data follow a similar trend, further validating our TGA analysis.

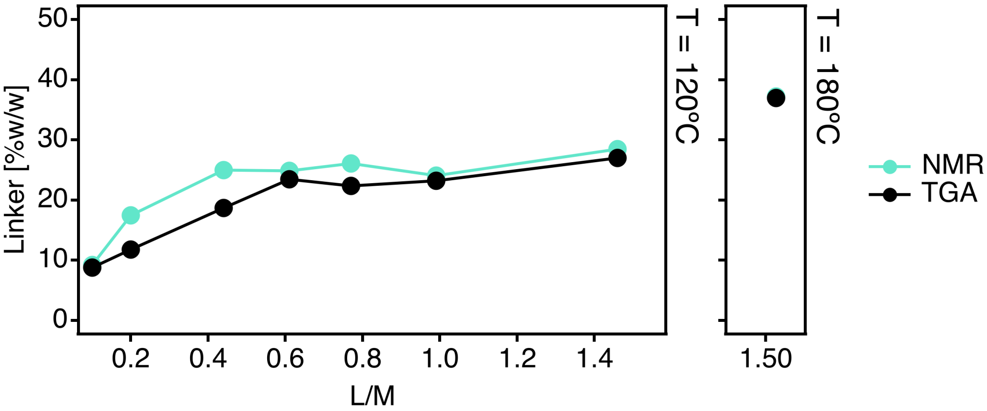

Supplementary Figure 16: Linker %w/w as calculated from TGA and NMR analysis

We considered FA and Cl, coming from the starting $HfCl_4$ metal salt, as possible capping ligands. FA/BDC and Cl/Hf ratios and the number of linkers per cluster (L) were obtained from ^{1}H-NMR, EDX and TGA data, respectively. The total cluster connectivity number (CN) of each sample can be calculated as:

$$Total\ CN = 2 \cdot L + \frac{FA}{BDC} \cdot L + 6\frac{Cl}{Hf}$$

As can be seen in Figure 17, total CN≈12 for all the samples studied, strongly suggesting that our samples are formed by Hf_6 nodes coordinated to linker BDC and modulator FA molecules and a minimum amount of chlorine atoms.

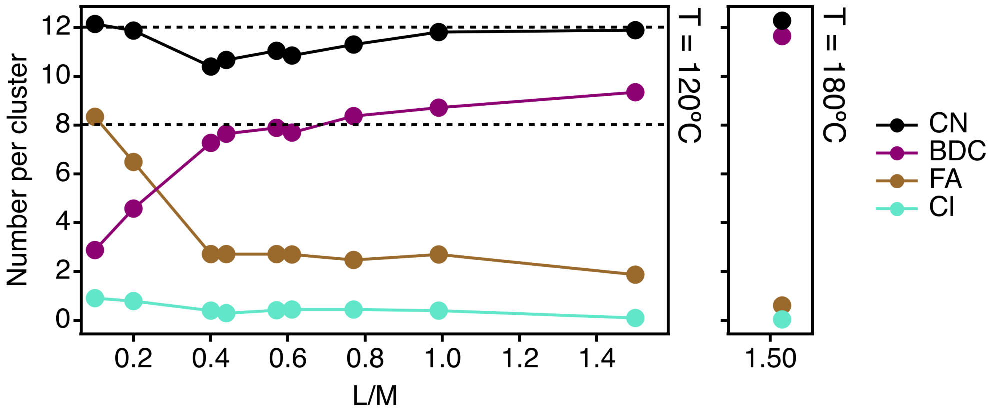

Supplementary Figure 17: Total cluster connectivity number and number of coordinated species per cluster as calculated from TGA, NMR and EDX analysis. Dotted lines are a guide for the eye for 12-connected ***fcu*** and 8-connected ***reo*** topologies

5 Imaging of defect domains with high-resolution electron microscopy

Figure 18 to Figure 21 correspond to Cs-corrected STEM images of different Hf-UiO-66 frameworks obtained. When both ***fcu*** and ***reo*** domains are present in the same particle they have been signaled with blue and red arrows, respectively. Moreover, the surface reconstruction present in some of the images has been highlighted with yellow arrows. We do not know what the exact nature of those surface reconstructions might be at the moment. Possible candidates could be related to ***hcp***, ***hns*** or ***hxl*** structures,[S4] or Hafmium formate.[S5] To try to get more information on this hypothesis, we are currently trying to obtain information along different zone axes, but it is resulting very challenging due to the morphology of the particles and their preferential [110] zone axis.

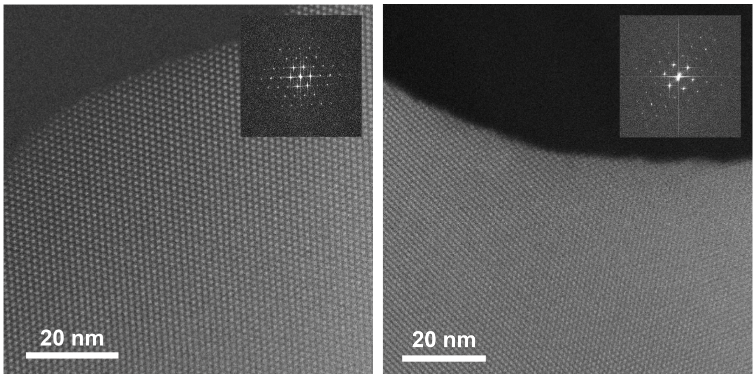

Supplementary Figure 18: Cs-corrected STEM analysis of a T = 180°C, L/M = 1.5 Hf-UiO-66 sample.

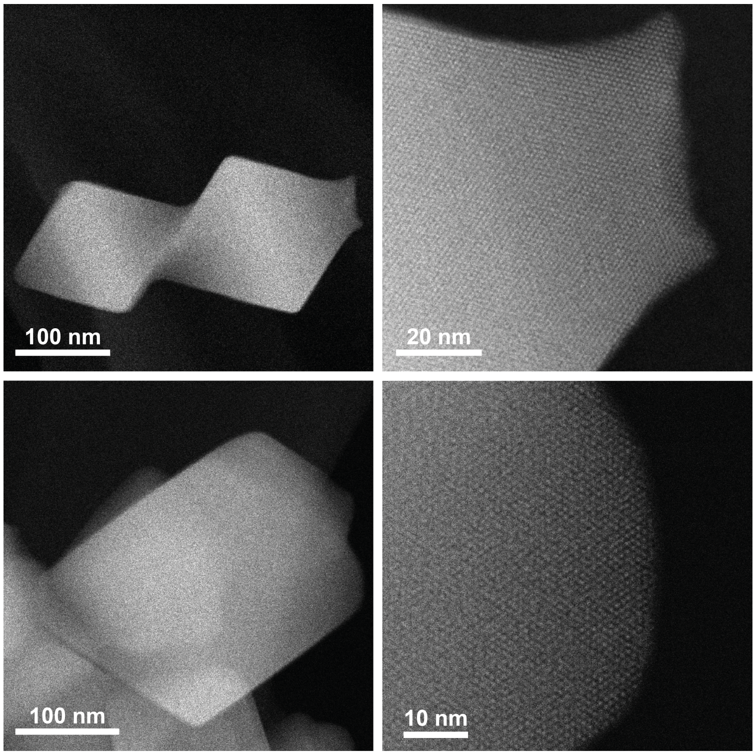

Supplementary Figure 19: Cs-corrected STEM analysis of a T = 120℃, L/M = 1.5 Hf-UiO-66 sample.

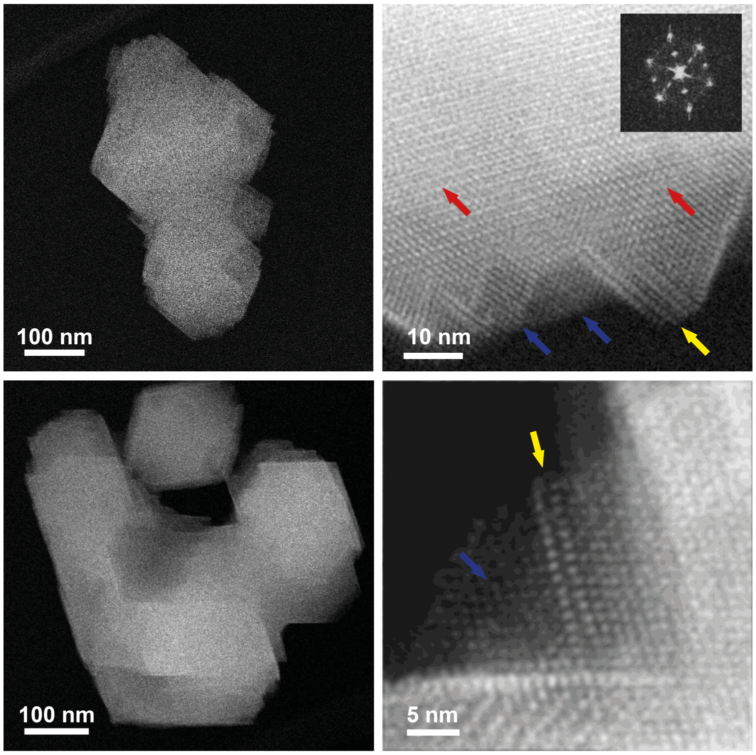

Supplementary Figure 20: Cs-corrected STEM analysis of a T = 120℃, L/M = 0.4 Hf-UiO-66 sample.

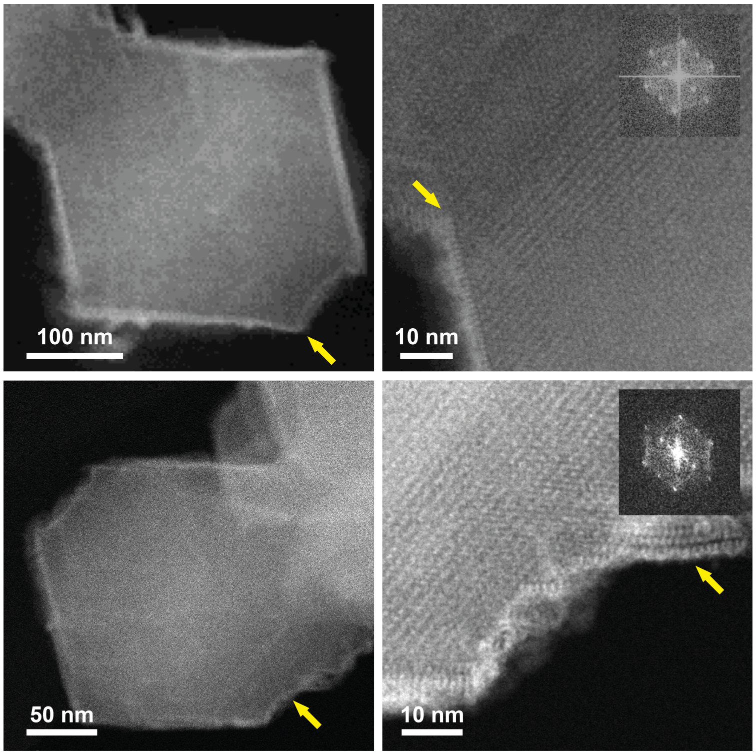

Supplementary Figure 21: Cs-corrected STEM analysis of a T = 120°C, L/M = 0.1 Hf-UiO-66 sample.

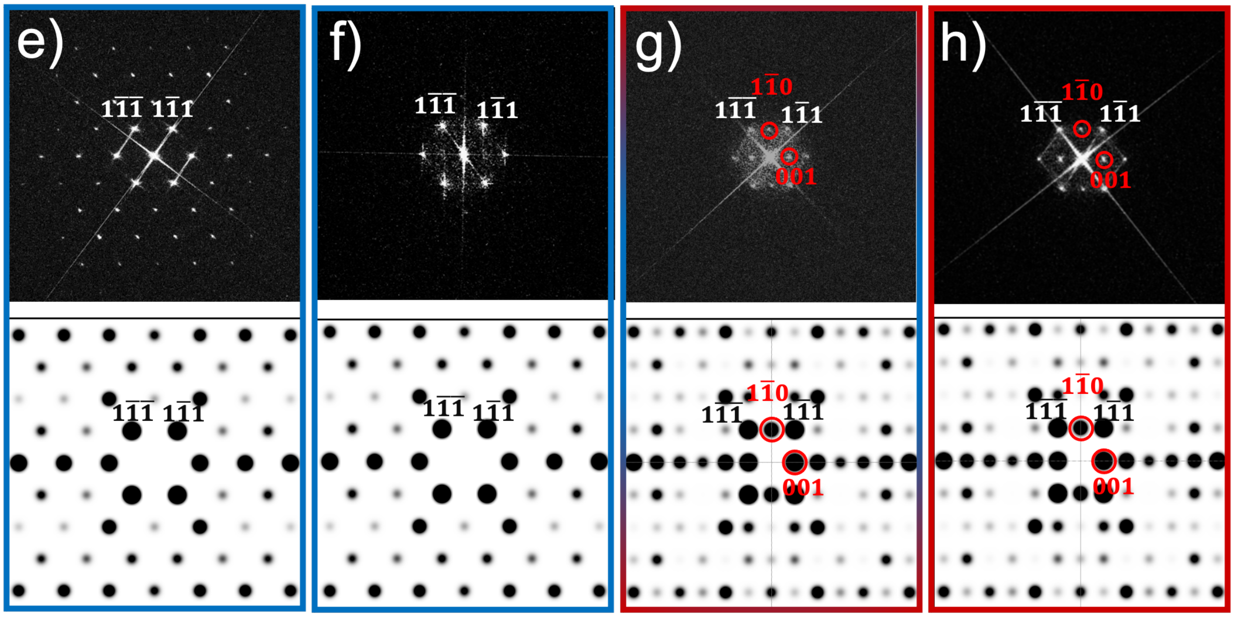

Supplementary Figure 22: FFTs and simulated ED patterns corresponding to Figure 6a-d represented at the same scale.

Figure 23 displays the Fast Fourier Transform (FFT) obtained from different areas of changing size of the same crystal revealing the presence of small amounts of sparse MC, in accordance with a sample with cluster connectivity close to 8.

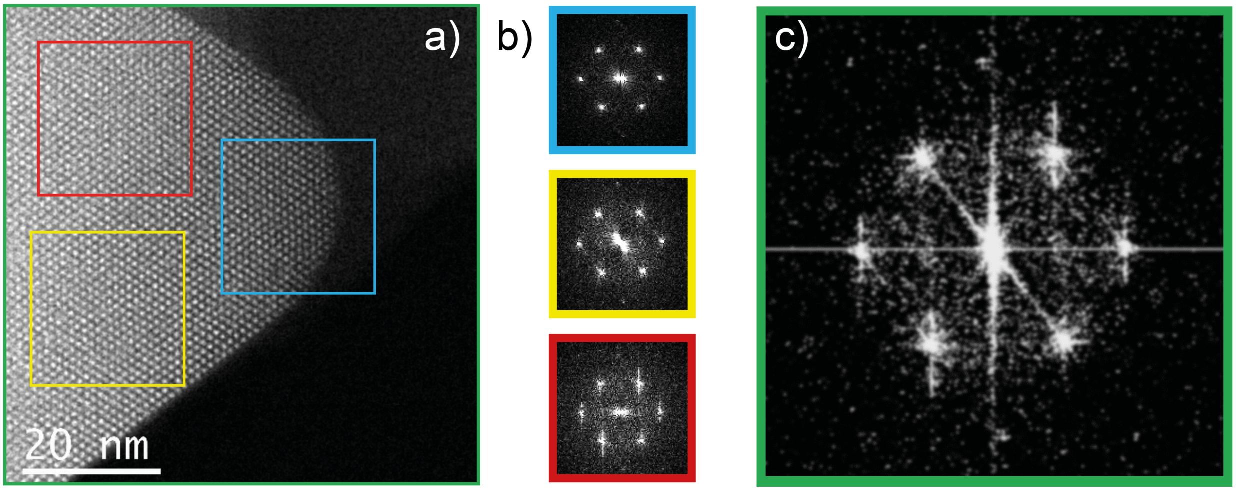

Supplementary Figure 23: a) Cs-corrected STEM-ADF low-magnification image of the T = 120°C, L/M = 1.5 sample displayed in Figure 6b. b) Small area FFT of the zones marked in (a) as coloured squares. c) Large area FFT.

Figure 24 displays the FFT obtained from two different areas of the same crystal revealing the differences that are observable through high-resolution imaging. The green square corresponds to a rich ***fcu*** phase (Figure 24b) whose FFT is shown inset and indexes as $Fm\bar{3}m$ space group. On the other hand, the blue square exhibits a different framework features (Figure 24c) and its correspondent FFT can be indexed as $Pm\bar{3}m$. The almost negligible reo spatial frequencies in b (white arrows) are owed to that fact that to retrieve clear enough FFT we selected an image area including several unit cells, inevitably also picking some small reo regions.

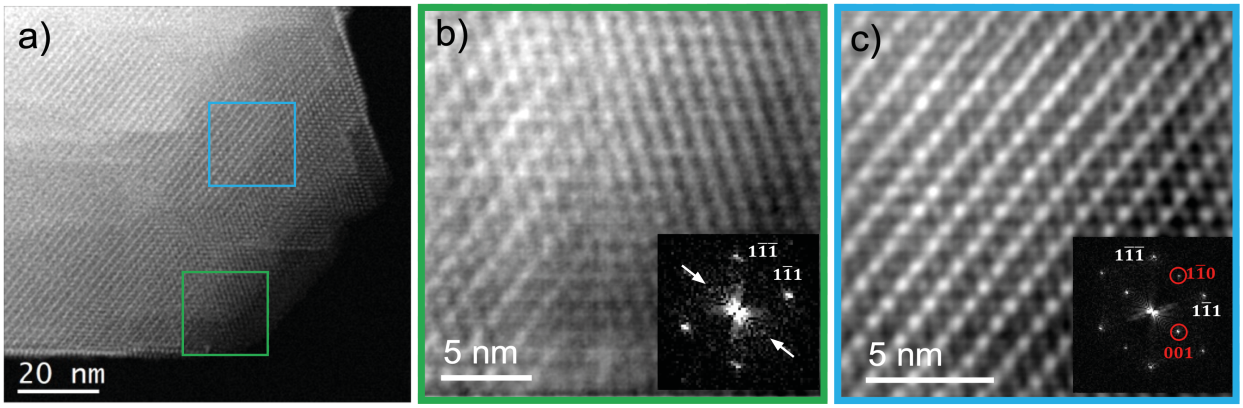

Supplementary Figure 24: Cs-corrected STEM-ADF analysis of T = 120℃, L/M = 0.4 particle displayed in Figure 6c. a) Low-magnification image of the framework. b) High-magnification image of the domain corresponding to ***fcu*** phase with the FFT inset, $Fd\bar{3}m$. c) High-magnification image of the domain corresponding to the ***reo*** phase with the FFT inset, $Pm\bar{3}m$.

6 Effect of defect concentration on catalytic performance

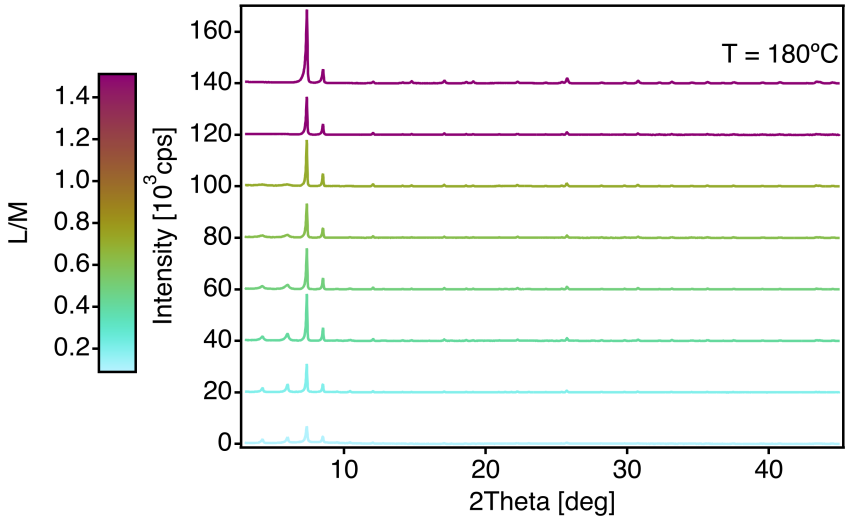

Supplementary Figure 25: PXRD as a function of the L/M ratio for a set of selected Zr-UiO-66 samples synthesized at T = 120°C, unless otherwise specified.

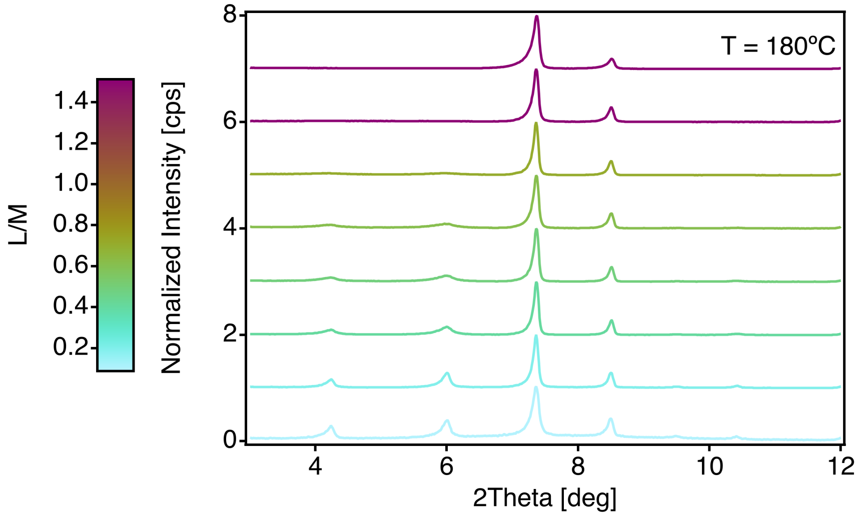

Supplementary Figure 26: Normalized PXRD as a function of the L/M ratio for a set of selected Zr-UiO-66 samples synthesized at T = 120°C, unless otherwise specified.

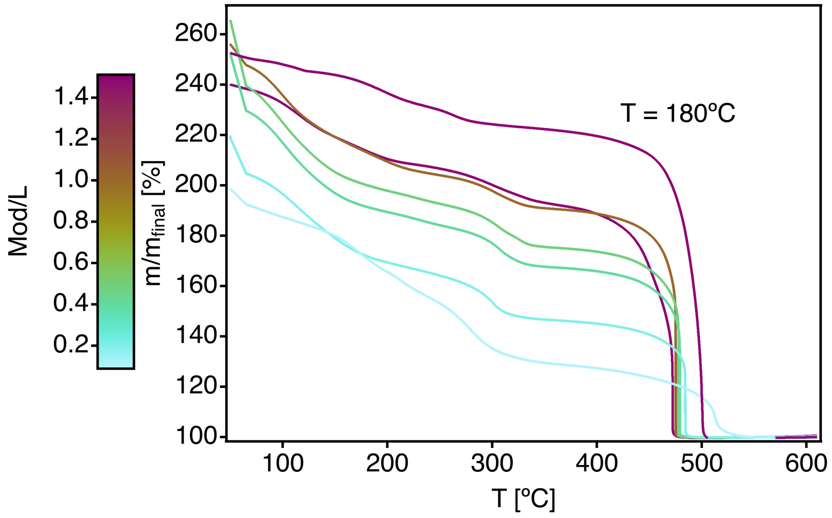

Supplementary Figure 27: TGA as a function of the L/M ratio for a set of selected Zr-UiO-66 samples synthesized at T = 120°C, unless otherwise specified.

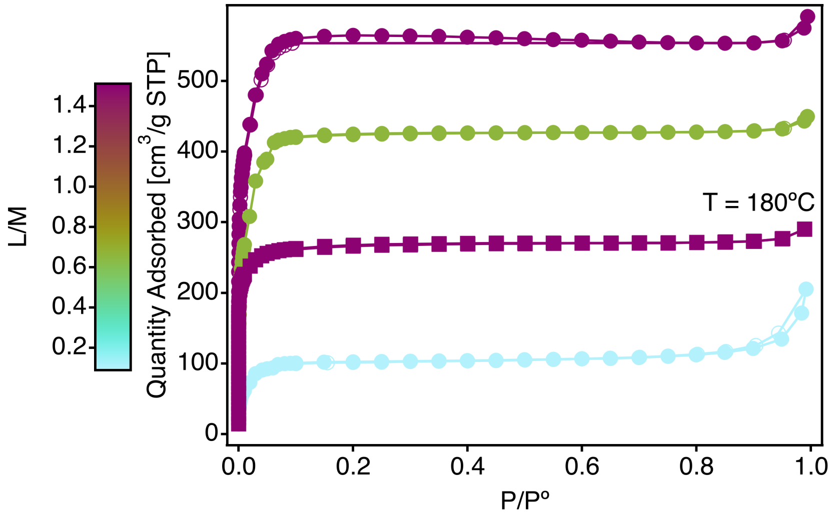

Supplementary Figure 28: N_2 isotherms as a function of the L/M ratio for a set of selected Zr-UiO-66 samples synthesized at T = 120°C, unless otherwise specified.

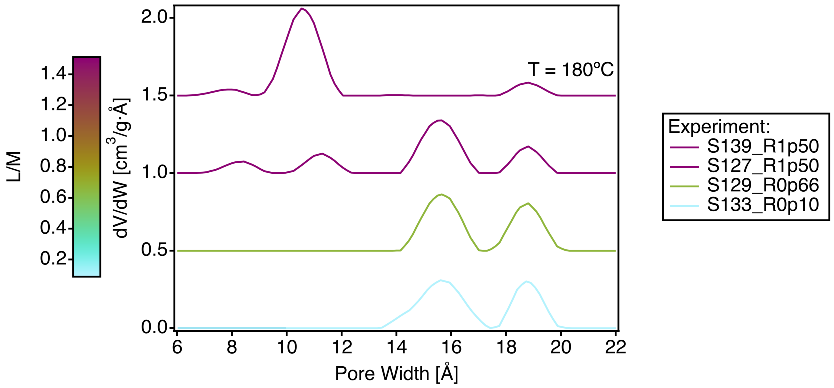

Supplementary Figure 29: PSD as a function of the L/M ratio for a set of selected Zr-UiO-66 samples synthesized at T = 120°C, unless otherwise specified.

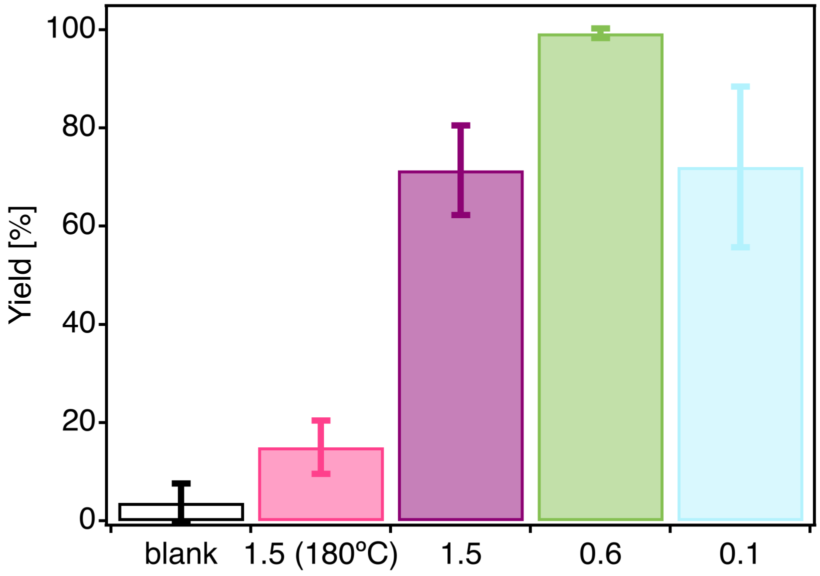

Supplementary Figure 30: Ring-opening amination yield measured after 10 hours of reaction for a defect-free sample used as a reference (L/M = 1.5, T = 180°C) and defective samples synthesized at 120°C for varying L/M ratios. Black sample correspond to a control sample in absence of MOF.

7 Supplementary Notes

Supplementary Note 1: We constated that I_{P2}/I_{P3} and I_{P1}/I_{P3} follow roughly the same trend with linker concentration. However, at very low reo fractions P1 tends to be wider than P2. Moreover, background contribution to the overall diffraction pattern is more intense at low angles making more difficult to measure I_{P1} than I_{P2}. That is why we decided to choose I_{P2}/I_{P3}.

Supplementary Note 2: Technical data on this procedure can be found in: https://www.wavemetrics.com/news/planar-triangulations and Igor Pro V9 Manual (https://www.wavemetrics.net/doc/IgorMan.pdf, ImageInterpolate, page V-380).

Compared to single-crystal data, in powder diffraction data all the reflections are collapsed in a 1D scan and averaged over all possible crystal orientations (from the measurement of many randomly oriented crystallites at once), and, therefore, 3D information is lost. This is translated in that refinements against powder diffraction data are more limited in their ability to determine precise atomic arrangements within the crystal structure . Furthermore, the IUCr checkCIF validation procedure is optimized for single-crystal data whereas it only partly covers structural refinements against powder diffraction data. This is the reason why we observe many A- or B-level alerts in the checkCIF files of the structural models obtained from powder diffraction data reported in this work.[S6] The following notes explicitly provide justification for them.

Supplementary Note 3: **PLAT430_ALERT_2_A**: This is due to disordered water molecules in the structure pores, which are expected to interact via hydrogen bonds. However, it is not possible to find the H positions from powder diffraction data.

Supplementary Note 4: PLAT602_ALERT_2_A: The crystal structure corresponds to a Metal-Organic-Framework, so it is expected that there will be large voids in it.

Supplementary Note 5: PLAT342_ALERT_3_B: The structure has been refined from multiphase powder diffraction data. Due to this, the complexity of the structure and the big unit cell, it is expected that the precision on the C-C bonds will be low.

Supplementary Note 6: PLAT430_ALERT_2_B: This is due to disordered water molecules in the structure pores, which are expected to interact via hydrogen bonds. However, it is not possible to find the H positions from powder diffraction data.

Supplementary References

(S1) Feng, X.; Jena, H. S.; Krishnaraj, C.; Leus, K.; Wang, G.; Chen, H.; Jia, C.; Voort, P. V. D. Generating Catalytic Sites in UiO-66 through Defect Engineering. *ACS Applied Materials & Interfaces* **2021**, *13*, 60715–60735.

(S2) National Institute of Standards and Technology NIST Chemistry WebBook, SRD 69. `https://webbook.nist.gov/chemistry/`.

(S3) Valenzano, L.; Civalleri, B.; Chavan, S.; Bordiga, S.; Nilsen, M. H.; Jakobsen, S.; Lillerud, K. P.; Lamberti, C. Disclosing the Complex Structure of UiO-66 Metal Organic Framework: A Synergic Combination of Experiment and Theory. *Chemistry of Materials* **2011**, *23*, 1700–1718.

(S4) Firth, F. C. N.; Cliffe, M. J.; Vulpe, D.; Aragones-Anglada, M.; Moghadam, P. Z.; Fairen-Jimenez, D.; Slater, B.; Grey, C. P. Engineering new defective phases of UiO family metal–organic frameworks with water. *Journal of Materials Chemistry A* **2019**, *7*, 7459–7469.

(S5) Liang, W.; Babarao, R.; Murphy, M. J.; D'Alessandro, D. M. The first example of a zirconium-oxide based metal–organic framework constructed from monocarboxylate ligands. *Dalton Transactions* **2015**, *44*, 1516–1519.

(S6) Spek, A. L. checkCIF validation ALERTS: what they mean and how to respond. *Acta Crystallographica Section E* **2020**, *76*, 1–11.
